# Complementary feeding patterns of Filipino infants and toddlers lack diversity, especially among children from poor households

**DOI:** 10.1186/s40795-020-00376-1

**Published:** 2020-10-26

**Authors:** Emma F. Jacquier, Imelda Angeles-Agdeppa, Yvonne M. Lenighan, Marvin B. Toledo, Mario V. Capanzana

**Affiliations:** 1 Nestlé Research, Vers-Chez-Les-Blanc, 1000 Lausanne, Switzerland; 2 Department of Science and Technology, Food and Nutrition Research Institute, Manila, The Philippines

**Keywords:** Complementary feeding, Infants, Children, Philippines

## Abstract

**Background:**

Consumption of nutritionally adequate complementary foods is essential for optimal growth and development of infants and toddlers, including those in developing countries. The aim of this study was to describe the food and beverage consumption patterns among 6–23.9 month old Filipino infants and toddlers, by household wealth.

**Methods:**

Data from 1087 infants and toddlers from the 2013 National Nutrition Survey were included. Dietary intake data was assessed using a 24Hr recall and population food intakes were stratified into pre-defined wealth categories.

**Results:**

Breast milk, infant formula, powdered milk and rice were the most commonly consumed foods and beverages across the age groups. Several differences in complementary feeding by wealth status were observed. Infants from poor households (69%) reported significantly greater consumption of human milk, than those from rich households (42%) who reported a significantly greater consumption of infant/toddler formula (Poor: 22%, Rich: 56%) (*P* < 0.05). A higher percentage of toddlers from rich households consumed protein-containing foods, cookies and cakes. There was a significant difference in vegetable consumption in 12–17.9 month old children (Poor: 17%, Rich: 31%; *P* = 0.021). Human milk and formula were the top contributors to energy in 6–17.9 month old children, while rice was the top energy contributor in 18–23.9 month old children.

**Conclusion:**

Milk and rice were the main dietary components in all Filipino children, contributing up to 60% of energy in the infants from poorer households. Consumption of protein-containing foods and vegetables were typically lower in poorer households. Interventions are required to enable caregivers of young Filipino children to provide complementary foods of high nutritional quality, particularly among children from the poor households.

## Key messages

A greater number of mothers from the poor households complied with the WHO recommendation to breastfeed, and children from the poor households were breastfed for longer than the rich households.

Milk consumption among older toddlers in the poor and middle wealth households decreased with age compared to toddlers from the rich households.

Consumption of nutrient poor foods is more common among infants and toddlers from the low wealth households compared to those from the rich households.

## Background

The nutritional quality of appropriate complementary foods is an important factor in the healthy growth and development of infants and young children [1]. During this period of early childhood, dietary habits and preferences begin to form, and caregivers must pay special attention to the choices and quality of foods offered to meet nutritional needs of the growing child. However, access to a variety of affordable and nutritious complementary foods may be challenging for households of limited means. Socioeconomic status has been recognised as a determinant of health [2] and individuals with higher socioeconomic status are more likely to have access to, and consume, healthier diets [3]. Hence, guidelines for complementary feeding aimed at low- and middle-income countries encourage feeding practices that promote consumption of nutrient dense foods [1].

Despite the economic growth of the Philippines over recent years, the National Nutrition Survey (NNS) reported that among infants aged 1 year old about 36.2% of children are stunted and that 20.8% are underweight [4]. There is also a high prevalence of anaemia (40.5%) in infants aged 6 months to less than 1 year [4]. Indeed, studies from the Philippines found that inadequate nutrient intakes in Filipino schoolchildren were more marked among those from poor families. Infants and young children in the Philippines also had a high prevalence of inadequate nutrient intakes [5].

At present, there has been no comprehensive assessment of socioeconomic differences in complementary feeding patterns in a nationally representative sample of Filipino infants and toddlers. Regional studies conducted to date have focused on sub-populations in Cebu and urban areas [6–10] and report sub-optimal nutrient intakes during the complementary feeding period. Furthermore, an association between complementary feeding practices and anaemia, stunting, iron and vitamin A deficiencies have also been identified [7]. The aim of this study was to describe the food and beverage consumption patterns among 6–23.9 month old Filipino infants and toddlers, and the associated impact on nutrient intakes, by household wealth status. This study provides novel results on the role of household wealth in determining nutrient adequacy in this vulnerable population.

## Methods

### Participants

The National Nutrition Survey (NNS) is a nationally representative, cross-sectional, epidemiological survey of the health and nutritional status of the Filipino population. The survey covers all 17 regions and 80 provinces of the country and applies a three-stage sampling system which enables full geographical coverage in both rural and urban areas. Survey response rate was high (87.7%) among the 35, 825 households surveyed. This study includes 1087 infants and young children aged 6–23.9 months who participated in the 2013 NNS in the Philippines. The protocol and survey instruments were approved by the Ethics committee of the Food and Nutrition Research Institute (FNRI) and informed consent was obtained from all households that participated. Anthropometric data (height, length and weight) were measured in the participant’s home, and the World Health Organization-Child Growth Standards were used to assess the nutritional status of the children, based on weight and height measurements. The socioeconomic status of the household was determined based on the possession of certain items e.g. vehicles, gadgets and household appliances. This data was then applied to classify households into wealth categories [4]. For the purpose of the food group analysis, the population was split into three categories; poor, middle and rich, however, for the in-depth analysis on usual nutrient intakes the poor and rich categories were further characterised into poor, poorest, rich and richest.

### Dietary intake data collection

A full description of the methods of the Filipino 2013 NNS has been previously described [4, 5). All dietary interviews were carried out in-person with the primary caregiver of the child. Trained dietitians collected data on all foods and beverages consumed using a structured 24 hour (Hr) dietary recall. A single 24 Hr recall is not reflective of habitual intakes of a person, however evidence suggests that two 24 Hr recalls in a proportion of the population can provide an approximate measure of habitual/long-term diet. Therefore, a second 24Hr recall was collected on a non-consecutive day from 50% of the households and was used to estimate usual nutrient intakes. Usual intakes were calculated using SAS software and the validated National Cancer Institute (NCI) method to calculate usual intakes was applied to calculate means and standard deviations [11, 12]. This method has been validated [13, 14] and is considered to be reflective of long term intakes within a given time period e.g. childhood. Amounts of foods and beverages were estimated using common household measures, and were converted into grams using a portion-to-weight list developed by FNRI for the survey.

The Individual Dietary Evaluation System (IDES), developed by FNRI, was used to process the dietary records and to estimate nutrient intakes from the 24Hr recalls [5]. Quality control of the data collected occurred at both the food level (amounts reported and coding to appropriate food groups) and at nutrient level to exclude implausible intakes [15]. Breast milk consumption was estimated using the child’s age and assigned volumes as in other national nutrition surveys of the infant population [16]. Foods were assigned to a food grouping system consisting of 85 food groups similar to those used in other dietary intake surveys of the infant and toddler population but adapted to include foods common in the local food culture [16].

### Statistical methods

The percentage of infants and young children consuming each food group was calculated by wealth category, as previously described [5]. The percent contribution of energy from food groups was calculated, at the population level, by summing the energy provided by each food group and dividing by the total energy intake by age and wealth category. Chi-squared tests were applied to examine the relationships between age, household wealth category and food group consumption. Usual nutrient intakes were estimated using the PC-SIDE software (Version 1.0) developed by Iowa State University (Ames, IA, USA) and data were analysed using Stata Version 13 (StataCorp, College Station, TX, USA). Since dietary variety increases at 12 months of age when children are eating more foods of the family table, the decision was taken to examine nutrient intakes starting from 12 months and also to examine, more closely, usual nutrient intakes by household wealth status.

## Results

The characteristics of the study population are presented in Table 1. The population was split almost equally by gender and age. In terms of wealth status the population was divided as follows; 50% from poor households; 20% from middle-class households and 30% from rich households. Additionally, approximately 54% of the children were from urban areas, and 46% resided from a rural setting. Approximately 5–6% of the children were underweight, 5–11% stunted, 2–4% wasted, and 4% overweight.

**Table 1:** Sample demographics of the 6–23.9 month Filipino study population

	Percent
Gender	
Boy	50.1
Girl	49.9
Age	
6–11.9 months old	33.0
12–17.9 months old	33.0
18–23.9 months old	34.0
Wealth categories	
Poor	50.0
Middle	20.1
Rich	29.9
Region	
Urban	54.1
Rural	45.9
Anthropometry	
Underweight	16.6
Stunting	22.8
Wasting	8.5
Normal weight	48.1
Overweight	3.9

### Milk types by age and household wealth category

The percent of consumers by milk types and by wealth category is presented in Table 2.

**Table 2:** Milk consumption (expressed as percent consuming) of Filipino 6–23 month olds, by wealth category

	6–11.9 mo				12–17.9 mo				18–23.9 mo			
	Poorest	Middle	Richest		Poorest	Middle	Richest		Poorest	Middle	Richest	
	*n =* 170	*n =* 71	*n* = 105	P^†^	*n =* 183	*n =* 68	*n* = 101	P^†^	*n = 175*	*n = 74*	*n = 114*	P^†^
All milk	96	100	98	0.162	91	93	97	0.204	70	81	95	**< 0.001**
Breast milk	69	63	42	**< 0.001**	54	44	24	**< 0.001**	35	27	19	0.061
Powdered milks	17	24	18	0.449	34	19	15	**0.001**	34	42	29	0.242
Formula	25	31	62	**< 0.001**	13	40	61	**< 0.001**	7	23	48	**< 0.001**
Infant	22	31	56	**< 0.001**	3	9	15	**0.001**	0	1	5	0.076
Toddler/preschooler	4	0	7	0.088	10	31	47	**< 0.001**	7	22	43	**< 0.001**

^†^Chi-square test for independence between wealth groups, *P* < 0.05 considered significant

Among 6–11.9 month old infants, there were significant differences by wealth status. Breast milk was consumed by 69% of infants from the poor households, compared to 42% in the rich households (*P* < 0.001). In contrast, infant formula was consumed by 22% of 6–11.9 month old infants from the poor households and 56% of children from rich households (*P* < 0.001). Breast milk was consumed by 54% of 12–17.9 month olds from the poor households, compared to 24% from the rich households (P < 0.001). Powdered milk was consumed by 34% of children from the poor households in this age group, compared to 15% from the rich households (*P* = 0.001). Toddler/preschooler formula was consumed by 10% of 12–17.9 month olds from the poor households, and 47% from the rich households (*P* < 0.001). In the older age group (18–23.9 month olds), the percentage of milk (all types) consumers in the poor and middle households were lower than those from rich households (*P* < 0.001). There was a wide variation in breast milk consumption in 18–23.9 month old children, ranging from 35% in children from the poor households to 19% from the rich households. Similar to the younger group, toddler/preschooler formula was consumed by 7% of 18–23.9 month olds from the poor households, compared to 43% from the rich households (P < 0.001).

### Food groups by age and household wealth category

There were notable differences in food group consumption by wealth status among 6–11.9 month old infants (Table 3). Infant cereal (grain-based cereal with nutrient fortification) was consumed by 7% of 6–11.9 month old infants from the poor households, compared to 23% of infants from the middle-class households and 19% from the rich households (*P* = 0.001). Among 12–17.9 month old children, consumption of eggs was greater in middle and rich households (*P* = 0.015). Consumption of cookies and cakes was higher in 12–17.9 month old children from rich households, while sugar sweetened beverage (SSBs) consumption was higher in infants from the poor households (*P* < 0.05). There was no significant difference in rice consumption between the age or wealth groups, however consumption of bread and noodles were greater among 12–17.9 month old toddlers from the rich households, compared to the poor households (P < 0.05). Protein-rich foods were more likely to be consumed by 12–17.9 month old toddlers from the rich households; pork (13% rich, 2% poor), sausages/luncheon meat (18% rich, 6% poor) and eggs (27% rich, 13% poor) (P < 0.05). Vegetable consumption was significantly greater among toddlers from the rich households (*P* = 0.021). While, percentage consumption of SSBs was greater among 12–17.9 month olds from the poor households (24%) compared to the rich households (13%) (*P* = 0.002). Similar consumption patterns of rice and grits were observed among the 12–17.9 month old and 18–23.9 month old toddlers. A greater percentage of 18–23.9 month old toddlers from richer households consumed sausages/luncheon meat compared to those from middle-income and poorer households (P = 0.002). There was an apparent difference in consumption of cookies (*P* = 0.017) and cakes (*P* = 0.028) also, with 38% of 18–23.9 month old toddlers from middle households consuming cookies and 18% of toddlers from the rich households consuming cakes.

**Table 3:** Food consumption (expressed as percent consuming) of Filipino 6–23.9 month olds, by wealth category

	6–11.9 mo				12–17.9 mo				18–23.9 mo			
	Poor	Middle	Rich		Poor	Middle	Rich		Poor	Middle	Rich	
	*n =* 170	*n =* 71	*n* = 105	P^†^	*n =* 183	*n =* 68	*n* = 101	P^†^	*n* = 175	*n* = 74	*n* = 114	P^†^
Rice	72	68	66	0.548	89	85	91	0.545	86	93	92	0.272
Grain-based mixed dishes	11	11	6	0.279	9	19	10	0.065	4	10	9	0.403
Corn grits	7	3	2	0.102	8	4	2	0.096	11	3	2	**0.014**
Infant cereal	7	23	19	**0.001**	0	2	4	**0.022**	0	0	0	–
Bread	7	4	12	0.118	11	18	25	**0.011**	23	30	27	0.639
Noodles	7	3	5	0.388	11	21	27	**0.011**	26	23	18	0.469
Vegetables	11	9	13	0.603	17	27	31	**0.021**	34	31	27	0.630
Fruits	9	7	2	0.071	14	10	15	0.621	16	14	18	0.741
Fish	12	9	8	0.484	29	34	21	0.146	45	41	33	0.247
Eggs	11	6	11	0.372	13	22	27	**0.015**	18	16	25	0.314
Sausages/luncheon meats	4	4	4	0.988	6	13	18	**0.009**	6	12	25	**0.002**
Pork	4	1	6	0.363	2	7	13	**0.001**	8	12	18	0.206
Chicken	2	1	2	0.969	6	7	12	0.172	7	10	16	0.186
Cookies	21	21	35	**0.022**	24	32	24	0.360	18	38	22	**0.017**
Sugar sweetened beverages (SSBs)	9	3	5	0.159	24	6	13	**0.002**	20	24	23	0.813
Table sugar	2	1	2	0.969	4	4	2	0.661	5	8	6	0.824
Crackers	7	17	6	**0.020**	18	19	21	0.871	18	19	14	0.589
Cakes	2	6	10	**0.007**	9	4	12	0.209	5	8	18	**0.028**

^†^Chisquare test for independence between wealth groups, *P* < 0.05 considered significant

### Total daily energy intakes by age and household wealth category

**Fig. 1:**
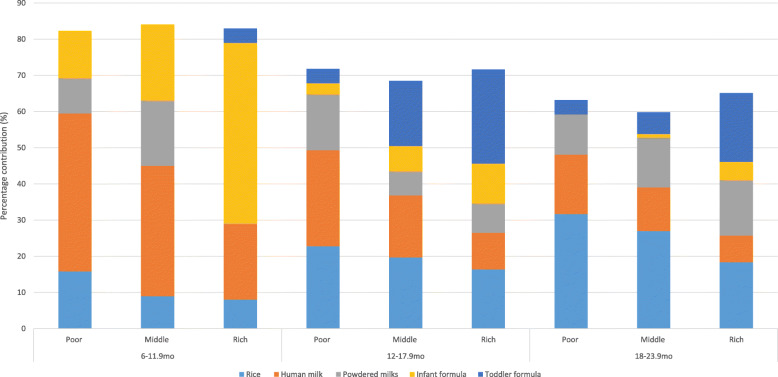
Top 5 contributors to energy intakes in Filipino infants and toddlers

The contribution of food to daily energy intakes by wealth status are presented in Fig. 1. Among 6–11.9 month olds, breast milk was the greatest contributor to energy intakes in infants from the poor households (44%). In contrast, infant formula provided the greatest energy contribution in 6–11.9 month old infants from the rich households (50%). Rice provided 16% of energy intakes in poor households, compared to 8% in infants from rich households, while infant cereal provided greater contributions in 6–11.9 month old infants from rich households than the poorer households.

Similar to the 6–11.9 month old infants, breast milk (27%) and rice (23%) were the greatest contributors to energy intakes in 12–17.9 month old toddlers from the poor households, while toddler/preschooler formula (26%) and rice (16%) were the greatest contributors to energy intakes in toddlers from rich households. Powdered milk contributed to 16% of energy intakes in 12–17.9 month old toddlers in the poor households, compared to 8% of intakes in the rich households, and infant formula contributed to 11% of energy in the rich households, compared to 3% in the poor households. Rice provided the greatest energy contributions in 18–23.9 month old toddlers from the poor households (32%), with toddler formula providing the greatest contributions in the rich households (19%). Breast milk (16%) and powdered milks (11%) were the second and third highest contributors in the poor households, compared to rice (18%) and powdered milks (15%) in the rich households. Bread and noodles contributed to energy intakes across the three wealth groups.

### Usual nutrient intakes in 12–23.9mo toddlers by household wealth category

The usual nutrient intakes of 12–23.9mo toddlers are presented in Table 4. Protein and total fat intakes were higher in toddlers from the rich and richest households, both in grams/day and as a percentage of total energy. Intakes of vitamin C, riboflavin, niacin, vitamin B_6_, folate, vitamin B_12_, vitamin D and vitamin E were higher among toddlers from the wealthiest households. Similarly, intakes of calcium, iron, magnesium and potassium were higher in toddlers from the richest households.

**Table 4:** Usual nutrient intakes in a subpopulation of Filipino 12–23.9 month old children, by wealth category

	Poorest	Poor	Middle	Rich	Richest
	*n =* 208	*n =* 141	*n =* 139	*n =* 116	*n =* 92
Carbohydrate (g/d)	108.2 ± 3.3	109.2 ± 4.1	112.4 ± 4.2	114 ± 4.3	116 ± 4.4
Total sugars (g/d)	31.3 ± 1.3	33 ± 1.6	30.8 ± 1.5	33.7 ± 2.6	29.3 ± 2.5
Dietary fiber (g/d)	2.3 ± 0.1	2.2 ± 0.1	2.3 ± 0.1	2.4 ± 0.1	2.4 ± 0.2
Protein (g/d)	19 ± 0.6	22.8 ± 1	21.8 ± 0.8	27.5 ± 1.3	27.7 ± 1.4
Total fat (g/d)	19.7 ± 0.6	25 ± 1	24 ± 0.9	30.5 ± 1.5	31.6 ± 1.5
SFA (g)	8.7 ± 2.6	8 ± 0.4	8 ± 0.4	9.4 ± 0.5	8.1 ± 0.6
MUFA (g)	5.6 ± 0.2	8.4 ± 0.6	6.4 ± 0.4	9.7 ± 0.8	6.9 ± 0.6
PUFA (g)	1.7 ± 0.1	2.7 ± 0.2	2.1 ± 0.1	2.9 ± 0.2	2.2 ± 0.2
**As percentage of total energy**					
Carbohydrate (%)	62.5 ± 0.7	58 ± 0.7	59.8 ± 0.6	55.2 ± 0.9	54.5 ± 0.9
Protein (%)	11.2 ± 0.2	11.8 ± 0.2	11.4 ± 0.2	12.8 ± 0.3	12.6 ± 0.3
Total Fat (%)	26.2 ± 0.7	29.9 ± 0.6	29.7 ± 0.6	31.9 ± 0.8	32.6 ± 0.7
**Vitamins**					
Vitamin A (μgRE/d)	195.7 ± 12.2	315 ± 31.1	197.1 ± 18.6	335.4 ± 40	159.8 ± 27.2
Vitamin C (mg/d)	21.2 ± 1	23.7 ± 1.8	32.2 ± 2.9	37.7 ± 3	55.7 ± 4.4
Thiamine (mg/d)	0.3 ± 0.02	0.4 ± 0.02	0.4 ± 0.03	0.5 ± 0.03	0.7 ± 0.05
Riboflavin (mg/d)	0.5 ± 0.03	0.8 ± 0.07	0.7 ± 0.1	1.1 ± 0.1	1.2 ± 0.1
Niacin (mg/d)	4.2 ± 0.1	4.4 ± 0.2	5.3 ± 0.3	5.8 ± 0.3	7.7 ± 0.5
Vitamin B6 (mg)	0.3 ± 0.02	0.6 ± 0.1	0.5 ± 0.03	0.6 ± 0.05	1.1 ± 0.1
Folate (DFE μg)	92.9 ± 6.2	85.4 ± 4.7	118.6 ± 9.3	107.8 ± 8.2	161.9 ± 14
Vitamin B12 (mg)	0.9 ± 0.04	0.8 ± 0.1	1.1 ± 0.1	1.2 ± 0.1	1.6 ± 0.1
Vitamin D (mg)	1.2 ± 0.1	1.4 ± 0.2	2.5 ± 0.2	2.7 ± 0.3	5.2 ± 0.7
Vitamin E (mg)	1.5 ± 0.1	1.5 ± 0.1	2.5 ± 0.3	2.1 ± 0.2	3.6 ± 0.4
**Minerals**					
Calcium (mg/d)	291.4 ± 16.5	447.3 ± 35.1	413.8 ± 30	632.9 ± 52.7	768.2 ± 62.7
Phosphorus (mg/d)	311.7 ± 13.4	453.2 ± 30.5	389.8 ± 21.8	544.1 ± 40.1	491.6 ± 33.1
Iron (mg/d)	3.2 ± 0.1	3.5 ± 0.2	4.6 ± 0.3	5.7 ± 0.4	9 ± 0.8
Sodium (mg/d)	469.5 ± 23.7	504.6 ± 21.2	474.2 ± 27	576.7 ± 32.4	555.7 ± 33.9
Zinc (mg)	3.1 ± 0.2	5.3 ± 1	3.8 ± 0.2	3.7 ± 0.2	5.2 ± 0.4
Magnesium (mg)	49 ± 1.9	49.2 ± 2.3	57.5 ± 3	63.5 ± 4.2	73.4 ± 4.8
Potassium (mg)	456.5 ± 153	441.8 ± 16.1	562.7 ± 25.7	606.3 ± 45.6	693.1 ± 47
Selenium (mg)	31.2 ± 1.3	27.3 ± 1.3	32.5 ± 1.7	31.8 ± 1.6	30 ± 1.9

## Discussion

The objective of this analysis was to describe the food and beverage consumption patterns among 6–23.9 month old Filipino infants and toddlers, and the associated impact on nutrient intakes, by household wealth status. The analysis highlights differences in food group consumption patterns, according to wealth status and provides insight into complementary feeding behaviours that may drive inequities in nutrient intake in infants and toddlers in the Philippines.

The prevalence of breastmilk consumption in this population has been previously reported by Denney et al. as 60% in 6–11.9 month old infants and 37% of 12–23.9 month old toddlers [5]. This analysis showed that there was higher breastmilk consumption among infants and toddlers from poorer households (Infants; Poor: 69%, Rich: 42%), and as a result breastmilk had a greater contribution to energy among these infants and toddlers (Infants; Poor: 44% Rich: 21%). Overall, the prevalence of breastfeeding in Filipino infants is sub-optimal and does not adhere to the guidelines of continued, on-demand breastfeeding until 2 years of age and beyond [1]. Nonetheless, the situation is better than that reported in national surveys from other geographies e.g. China (44% 0–5.9mo) [17] and Mexico (15% 0–3.9mo) [18]. Public health strategies, including educational campaigns and the availability of lactation consultants, are required to further increase the rates of breastfeeding among Filipino mothers.

In the current study, breast milk contributes 7–40% of energy intakes of infants and toddlers between 6 and 24 months, depending on the child’s stage and wealth status, with infant/toddler formula providing from 1 to 50% of energy intakes. Additionally, rice contributed from 8 to 23% of energy intakes in this population. However, breast milk and rice are low in minerals such as iron and zinc, and infant need complementary foods, in particular, those that provide iron, zinc and calcium, from the age of 6 months [19, 20]. Infants typically consume relatively small amounts of complementary foods between 6 and 11.9 months, therefore, the nutrient density of these foods needs to be high [21]. The current analysis suggests that consumption of nutrient dense food at this age is low, therefore educational strategies are needed to inform mothers of the importance of consuming nutrient dense foods at this early life stage. An extensive study examining Filipino infant and child feeding (6–23 months of age) in urban areas reported that children who are breastfed were more likely to be anaemic or iron deficient after 6 months of age than those receiving fortified foods [7]. This is similar to results reported in other countries including India, China and Japan [22–24]. Fortified young child beverages provide a source of iron among Filipino infants (6–11.9mo) and toddlers (12–23.9mo), respectively [5]. However, consumption of these products was more prevalent in infants and toddlers from higher income households. This may, in part, explain some of the differences in nutrient intakes according to household wealth category.

Rice is the most commonly consumed (94.8%) food in the Philippines, and is also the primary source of energy, protein, iron, thiamin and niacin [4]. In the current study the percentage contribution of rice to total daily energy, was consistently higher among children from the poorest households in infants and toddlers. This was reflected by poorer intakes of vitamins and minerals in toddlers from poorer backgrounds. However, a recent paper by Denney et al. in the same population, identified inadequate of protein and B vitamins in Filipino infants and toddlers at population level [5]. Ideally, the mandatory rice with vitamins and minerals in the Philippines could provide a public health opportunity to improve nutrient intakes, particularly among children from poorer households. For example, a study among school kids revealed significant reduction in anaemia after a 120 day supervised fortified lunch feeding program [25]. Unfortified complementary foods that are plant-based generally do not provide sufficient key nutrients such as iron, zinc and calcium in adequate amounts for very young children in developing countries [26]. The current analysis identified low intakes of iron, zinc and calcium in young Filipino children, particularly in those from poor households. Therefore, initiatives to support fortification of commonly consumed foods could help to close this gap in nutrient intakes.

In general, infant cereal consumption is low in the Philippines, with the exception of a sub-population from the rich households. In the current study, iron-fortified infant cereal, as recommended in complementary feeding by the American Academy of Paediatricians [27], was lacking from the diet, except among 6% of 6–11.9mo infants from the middle and rich households. The current study identified a very limited diversity of foods in the diets of Filipino infants and children, with less than 5 foods making up 80% of the diet in 6–11.9 month olds and 60% of the diet in 18–23.9 month olds. Nutrient-dense protein foods such as eggs, pork and chicken were more likely to be consumed by children from richest households. Whereas, fish was more likely to be consumed by children from low wealth households. Improving access to nutritious or fortified foods for children, particularly breast-fed infants such as infant cereal, could help to improve micronutrient intakes. However, it must be noted, that even in the U.S., iron and zinc were identified as problem nutrients [16] despite the widespread availability of fortified foods for infants. Therefore, ensuring adequate nutrient supply in complementary feeding is clearly complex and requires further detailed study in order to examine *how* young children can meet their nutrient needs in these challenging circumstances.

In the current study, SSBs were more likely to be consumed by infants and young toddlers from the poorest households. However, SSBs were consumed at approximately equal prevalence across wealth categories in 18–23.9 month olds. Since the nutrient needs, according to body weight, of infants and young children are very high [27] there is typically no room in the diet for nutrient-poor foods such as SSBs. In the current study, infants were more likely to consume fruit juice and powdered chocolate milk than other types of SSBs. In order to help combat the nutritional inadequacies in the Philippines, fruit juice and chocolate milk are often fortified and have been shown to be among the top sources of micronutrients in the diets of Filipino children [5]. Fortified fruit juice has been effective at reducing the basal level of iron deficient anaemia in Filipino school children from 100 to 13% [28, 29]. However, it must be noted that unpublished data from the most recent Philippines National Nutrition Survey shows that the prevalence of overweight has increased in 3–5 year old children between 2015 and 2018, with a similar trend observed in school-aged children (Preliminary Results National Nutrition Survey 2018, Food and Nutrition Research Institute). Evidence suggests that excess weight gain in infancy, of which consumption of a poor diet (low in fruit and vegetables and high in sugar) or a high calorie diet, in early childhood could be a contributing factor, has been associated with increased risk of developing obesity and associated comorbidities in later life [30–32]. The current study demonstrated that the diet of Filipino infants is rich in carbohydrates, with limited protein intakes and low adherence to the local Filipino *Pinggang Pinoy* recommendation for frequent consumption of fruit and vegetables. This may be due to a number of factors including high cost, limited access to fresh fruit and vegetables, poor nutrition knowledge and family members’ food preferences [33]. Therefore, the importance of fruit and vegetables, meat, poultry, fish and eggs, should be emphasised in Filipino households, regardless of wealth status. Public health authorities may wish to investigate methods to improve accessibility to such foods, particularly among low-income households.

This study presents a very comprehensive evaluation of complementary feeding patterns of Filipino infants and young children. It relied on nationally representative data from the National Nutrition Survey in 2013. Several limitations are noted. The dietary intake estimates for the infants and young children were based on reports by parents, and may have included inaccuracies leading to over- or under-estimation of food intakes. The data were collected in 2013, and it is possible that new foods on the market in the Philippines, especially fortified foods, could have resulted in subsequent improvements in the nutrient intake estimates of young children. Furthermore, we used a single 24 h recall to assess food group intakes, and this may not fully capture habitual dietary intakes. Dietary supplements were not included in the analysis and, if used, may have also increased nutrient intakes in this population.

## Conclusion

Strategies aiming to make fortified foods affordable and accessible to low-income populations, and the widespread use of vitamin and mineral supplements of young children and their breastfeeding mothers could help to improve nutrient intakes. This study provided a detailed assessment of nutrient intakes in this population according to wealth category, but additional assessments of foods available in the local food supply would be required to determine if food fortification or supplementation would be the most appropriate strategy for meeting nutrient gaps. In addition, environmental and conservational issues need to be considered when evaluating nutrient sources for low-income and vulnerable populations in the Philippines.

In light of the high rates of anaemia and inadequate nutrient intakes among Filipino infants, future research should focus on determining how best to meet the nutritional needs of young Filipino children. Further work is needed to identify potential targets for food fortification, and to clarify the impact of fortification (or supplementation) with single or multiple nutrients is needed. Diet modelling studies on the impact of fortification of commonly consumed foods or supplementation may shed light on the feasibility of potential strategies aimed at improving nutrient intakes, particularly among infants and toddlers from poor households.

## Data Availability

The datasets supporting the conclusions of the study are included in the article. Any additional data will be available on request. The datasets used and/or analyzed during the current study are available from the FNRI on reasonable request.

## Abbreviations

24Hr: 24 hour
NNS: National Nutrition Survey
FNRI: Food and Nutrition Research Institute
IDES: Individual Dietary Evaluation System
SSBs: Sugar-sweetened beverages
